# LncRNA MEG3 promotes melanoma growth, metastasis and formation through modulating miR-21/E-cadherin axis

**DOI:** 10.1186/s12935-019-1087-4

**Published:** 2020-01-10

**Authors:** Liangcai Wu, Lifei Zhu, Yanchang Li, Zhixin Zheng, Xi Lin, Chaoying Yang

**Affiliations:** 1 Department of Dermatology, The Sixth Affiliated Hospital, Sun Yat-sen University, No. 26, Yuancun ErHeng Road, Guangzhou, 510655 China; 2 Department of Pharmacology, Medical College, Jinan University, Guangzhou, 510632 China

**Keywords:** Melanoma, lncRNA MEG3/miR-21/E-cadherin regulatory axis, Development and progression of tumor

## Abstract

**Background:**

Melanoma is the most aggressive type of skin cancer with high mortality rate and poor prognosis. lncRNA MEG3, a tumor suppressor, is closely related to the development of various cancers. However, the role of lncRNA MEG3 in melanoma has seldom been studied.

**Methods:**

RT-PCR was used to examine the expressions of lncRNA MEG3 and E-cadherin in melanoma patients and cell lines. Then, the biological functions of lncRNA MEG3 and E-cadherin were demonstrated by transfecting lncRNA MEG3-siRNA, lncRNA MEG3-overexpression, E-cadherin-siRNA and E-cadherin-overexpression plasmids in melanoma cell lines. Moreover, CCK8 assay and colony formation assay were utilized to assess the cell proliferation; Transwell assay was performed to evaluate the cell invasive ability; and tumor xenografts in nude mice were applied to test the tumor generation. Additionally, the target interactions among lncRNA MEG3, miR-21 and E-cadherin were determined by dual luciferase reporter assay. Finally, RT-PCR and WB were further conducted to verify the regulatory roles among lncRNA MEG3, miR-21 and E-cadherin.

**Results:**

The clinical data showed that lncRNA MEG3 and E-cadherin expressions were both declined in carcinoma tissues as compared with their para-carcinoma tissues. Moreover, lncRNA MEG3 and E-cadherin expressions in B16 cells were also higher than those in A375 and A2058 cells. Subsequently, based on the differently expressed lncRNA MEG3 and E-cadherin in these human melanoma cell lines, we chose B16, A375 and A2058 cells for the following experiments. The results demonstrated that lncRNA MEG3 could suppress the tumor growth, tumor metastasis and formation; and meanwhile E-cadherin had the same effects on tumor growth, tumor metastasis and formation. Furthermore, the analysis of Kaplan–Meier curves also confirmed that there was a positive correlation between lncRNA MEG3 and E-cadherin. Ultimately, dual luciferase assays were further used to verify that lncRNA MEG3 could directly target miR-21 which could directly target E-cadherin in turn. Additionally, the data of RT-PCR and WB revealed that knockdown of lncRNA MEG3 in B16 cells inhibited miR-21 expression and promoted E-cadherin expression, but overexpression of lncRNA MEG3 in A375 and A2058 cells presented completely opposite results.

**Conclusion:**

Our findings indicated that lncRNA MEG3 might inhibit the tumor growth, tumor metastasis and formation of melanoma by modulating miR-21/E-cadherin axis.

## Background

Melanoma is the highest lethal cancer of the common skin cancers which is listed as the seventh most frequent malignant tumor in females and the fifth most frequent malignant tumor in males world wide and metastatic melanoma is the most aggressive form of this cancer [1, 2]. The incidence and mortality of melanoma have continued to increase steadily in industrialized Caucasian populations over the past decades [3, 4]. Epidemiological data clearly revealed that 74,100 individuals suffered from melanoma annually while 8700 die per year in the United States, so melanoma seriously affects human health [2, 5]. Despite the notable improvements in treatments made in recent years, the prognosis remains poor for advanced melanoma patients, mainly due to its high mortality with intrinsic resistance to chemotherapy or radiotherapy, aggressive clinical behavior and faster metastatic potentials [6]. For early-stage melanoma, many methods are used for the treatment of melanoma, including surgery, combined chemotherapy, radiotherapy and molecular targeted therapy, and meanwhile surgery is always recognized as the mainstay of treatment with 90% cure rates [7]. Therefore, early melanoma detection is the key to improving the survival and once the diagnosis is delayed, the mortality of melanoma would be rapidly elevated [7, 8]. However, it is very difficult for dermatopathologists in clinical work to determine the histopathologic types and tumor stages of melanoma in a subset of cases [8, 9]. Hence, better understanding of the underlying molecular mechanisms about malignant melanoma tumorigenesis and progression will be helpful to explore novel sensitive and specific biomarker or therapeutic agents.

Long non-coding RNA (lncRNA), defined as a group of transcripts with a length > 200 nucleotides with limited protein coding potential, have been implicated in the onset and development of different human cancers by chromatin remodeling, as well as transcriptional and post-transcriptional regulation [10, 11]. For example, lncRNA DLX6-AS1 promotes liver cancer by increasing the expression of WEE1 via targeting miR-424-5p [12]; lncRNA MORT overexpression inhibits cancer cell proliferation in oral squamous cell carcinoma by downregulating ROCK1 [13]; lncRNAGIHCG induces cancer progression and chemoresistance and indicates poor prognosis in colorectal cancer [14].It has been found that lncRNA maternally expressed gene 3 (MEG3) with tumor suppressor activity was frequently either lost, mutated or decreased level in many human tumors and tumor derived cell lines [15]. For instance, lncRNA MEG3 inhibits the progression of prostate cancer by modulating miR-9-5p/QKI-5 axis [16]; Down regulation of lncRNA MEG3 promotes colorectal adenocarcinoma cell proliferation and inhibits the apoptosis by up-regulating TGF-β1 and its downstream sphingosine kinase 1 (SPHK1) [17]; lncRNA MEG3 suppresses the tumorigenesis of hemangioma by sponging miR-494 and regulating PTEN/PI3K/AKT pathway [18]. Additionally, lncRNA MEG3 was also explored in the development of melanoma, e.g., lncRNAMEG3 suppresses the proliferation and invasion of melanoma by regulating CYLD expression mediated by sponging miR-499-5p [19]. However, previous studies have uncovered that miR-21 was strongly expressed in melanoma and was related to the degree of dedifferentiation and aggressiveness of melanoma [20]. Furthermore, miR-21 has been considered as a “mastermind of metastasis” in many cancers, such as melanoma, colon cancer, pancreatic cancer, breast cancer, lung cancer, gastric cancer [21]. On the other hand, bioinformatics analysis displayed that there might be a targeted regulatory interaction between lncRNA MEG3 and miR-21. Furthermore, based on the faster metastasis function of miR-21, E-cadherin, responsible for adhesion of melanocytes [22], was also discovered to be a target gene of miR-21. Therefore, in this study, we sought to look at the relationship among lncRNA MEG3, miR-21 and E-cadherin, and further investigate the possible role of lncRNA MEG3/miR-21/E-cadherin regulatory axis in progression of melanoma.

## Materials and methods

### Patients and specimens

A total of 25 melanoma patients who diagnosed without other severe diseases and mental health problems were recruited to the present study in the Sixth Affiliated Hospital, Sun Yat-sen University (Guangzhou, China) from January 2013 to December 2017. All patients, who didn’t receive treatment with any pre-surgery adjuvant therapies such as radiotherapy, chemotherapy, etc., received surgical resection, and tumor tissues and matched adjacent non-tumor normal tissues 5 cm away from the cancer lesion were gathered during the surgery. The histopathologic diagnosis was confirmed following the WHO criteria by two independent pathologists at least. Fresh tissue samples were immediately snap-frozen in liquid nitrogen and then stored at − 80 °C until use.

All of the patients who provided samples were well informed about the use of samples and informed consents were also signed. Meanwhile, the present study was approved by the Research Ethics Committee of the Sixth Affiliated Hospital, Sun Yat-sen University in accordance with the ethical guidelines of the Declaration of Helsinki.

### Cell line, culture conditions and cell treatment

Human melanoma cell lines (HEMn, A375 and A2058) and murine melanoma cell lines (B16) were obtained from the Type Culture Collection of the Chinese Academy of Sciences (Shanghai, China) and stored in the Central Laboratory of the Center for Experimental Medicine, Sixth Affiliated Hospital, Sun Yat-sen University (Guangzhou, China). B16 cells were cultured in Eagle’s minimum Essential medium (EMEM; Gibco, USA) containing 10% heat-inactivated fetal bovine serum (FBS; Gibco, USA) and 2.5% penicillin and streptomycin at 37 °C in a humidified atmosphere containing 5% CO_2_. The cells in the exponential phrase were used for experiments.

To enhance lncRNA MEG3 and E-cadherin exogenous expressions, the full lengths of lncRNA MEG3 and E-cadherin sequences were both synthesized by means of polymerase chain reaction (PCR) and inserted into pcDNA3.1 empty plasmid (GenePharma, China), termed as pcDNA3.1-lncRNA MEG3 and pcDNA3.1-E-cadherin, respectively. However, to attenuating lncRNA MEG3 expression and E-cadherin expression, siRNA against lncRNA MEG3 (lncRNA MEG3-siRNA) and siRNA against E-cadherin (E-cadherin-siRNA) were both generated by GenePharma, China. B16, A2058 and A375 cells were plated in 6-well plates at a density of 5 × 10^6^ cells/well and cultured to reach 80–90% confluence for transfection using Lipofectamine 2000 (Promega, USA) according to manufacturer’s instructions. The B16 cells were transfected with lncRNA MEG3-siRNA and E-cadherin-siRNA, while A2058 and A375 cells were transfected with pcDNA3.1-lncRNA MEG3 and pcDNA3.1-E-cadherin. In addition, in order to determine the target interaction between miR-21 and E-cadherin, we also set up the B16-miR21 + lncRNA MEG3, A2058-miR21 + lncRNA MEG3and A375-miR21 + lncRNA MEG3 groups.

### Total RNA preparation and real-time polymerase chain reaction (RT-PCR)

Total RNA was extracted from culture cells (including HEMn, A375, A2058 and B16 cells) using Trizol reagent (Thermo Fisher Scientific, USA) based on the manufacturer’s instructions. RNA samples were quantified using a BioPhotometer and the integrity of the RNA was verified by agarose-formaldehyde gel electrophoresis. Then, the complementary DNA (cDNA) was synthesized using the GoScriptTM Reverse Transcription system (Promega, USA) according to the manufacturer’s protocols. The expressions of lncRNA MEG3, E-cadherin and miR-21 were further examined using the SYBR-Green PCR Master Mix kit (TAKARA, Japan) on an ABI Prism 7900 Sequence Detection System (Applied Biosystems, USA). The reactions were incubated in a 96-well optical plate at 95 °C for 2 min, followed by 40 cycles of 15 s at 95 °C and 32 s at 60 °C and dissociation at 95 °C for 60 s, 55 °C for 30 s and 95 °C for 30 s. The sequences of the primers are listed as follows: human lncRNA MEG3 forward, 5′-GCTATGCTCATACTTTGACTC-3′ and reverse 5′-CATCATAAGGGTGATGACAG-3′; mouse lncRNA-MEG3 variant 1-wide type forward, 5′-CCGCTCGAGAGCCCCTAGCGCAGACGGCGGAG-3′ and reverse, 5′-ATAAGAATGCGGCCGCTTTTTGTTAAGACAGGAAACACATT-3′; mouse lncRNA-MEG3 variant 1-mutant forward, 5′-GGCCCTGTTGTTTAGTCTGGAATGAGCATGCTACTG-3′ and reverse, 5′-CATTCCAGACTAAACAACAGGGCCTTGGAGTTGCCAG-3′; E-cadherin forward, forward, 5′-TGCTACTTTCCTTGCTTCTG-3′ and reverse 5′-TCTCTGCCTCTTGAGGTAAC-3′; miR-21 forward, 5′-ACACTCCAGCTGGGTGTAAACATCCTACACTCT-3′ and reverse 5′-CTCAACTGGTGTCGTGGA-3′; 18S rRNA forward, 5′-CTCGCTTCGGCAGCACA-3′ and reverse 5′-GCGGCGCAATACGAATGCCCC-3′; and U6 snRNA forward, 5′-CTCGCTTCGGCAGCACA-3′ and reverse 5′-AACGCTTCACGAATTTGCGT-3′. The fold changes of these genes in the expressions were evaluated by2^−∆∆Ct^ method. mRNAs expression and miRNAs expression were normalized to endogenous control 18S rRNA and U6 snRNA, respectively. Each sample was assessed in triplicate.

### Cell Counting Kit (CCK)-8 proliferation assays

Cell proliferation was examined via a CCK-8 kit (Dojindo Molecular Technologies, Japan) following the manufacturer’s manual. In brief, 100 µl cell suspension of transfected cells was planted into 96-well plates at 24 h post-transfection. Following incubation for 0, 1, 2, 3, 4, 5, 6 and 7 d at 37 °C in an atmosphere containing 5% CO_2_, 10 µl of CCK-8 solution was added to each well for additional 2 h incubation. The optical density (OD) value was determined at a wavelength of 450 nm using a microplate spectrophotometer (BioTek Instruments, USA). Each assay was performed in triplicate and independently repeated three times.

### Colony formation assay

Transfected cells were seeded into 12-well plates at a density of 1000 cells/ml (2 ml per well). After 14 days culture, the cells were washed with PBS and then fixed with 3.7% methanol for 15 min and stained with 0.1% crystal violet for 30 min at room temperature. Clones with > 50 cells were counted and images of the colonies were captured by an inverted microscope (Leica, Germany).

### Cell invasion assay

Invasion was investigated with an in vitro Transwell Matrigel invasion assay (Corning, USA) which was made up of 24-well artificial basement membrane inserts that had an 8 µm precoated Matrigel membrane filter and allowed single cells to invade. 1 × 10^5^ transfected cell suspension was seeded into the upper chamber which is serum-free medium, while fresh EMEM media containing 10% FBS were supplemented into the lower chamber. After incubation for 48 h, the cells remaining on the upper membrane were carefully removed with cotton swabs. Meanwhile, 4% polyformaldehyde and 0.1% crystal violet were used to fix and stain the cells that had invaded through the membrane for 20 min and 15 min, respectively. Ultimately, the invasion ability was calculated by the number of cells that penetrated into the matrigel on the lower chamber using photographic images.

### Tumor xenografts in nude mice

The 3 to 5-week-old female BALB/c nude mice (5 mice per group) obtained from Beijing Vital River Experimental Animal Technology Co. Ltd (Beijing, China) were maintained under pathogen free condition. All experiments were known and approved by the animal center of the Sixth Affiliated Hospital, Sun Yat-sen University in accordance with the National Institute of Health’s (NIH) Animal Care and Use Committee guidelines.

The stably transfected cells (1.5 × 10^6^) were injected subcutaneously into the flanks of female BALB/c nude mice. The tumor size was measured using a caliper every 3 days from the 4th day after injection. The tumor volume was calculated using the following formula: volume = (length × width^2^)/2. The mice were sacrificed on day 44 and the tumors were separated for further analysis.

### Western blot (WB) analysis

Treated cells (including A375, A2058 and B16 cells)were lysed in cold radioimmunoprecipitation assay (RIPA) buffer containing 50 mM Tris–HCl (pH 7.5), 150 mM NaCl, 0.1% Nonidet P-40, and a mixture of protease inhibitors, and the protein concentration was quantified by Bradford Protein Assay Kit (ThermoFisher Scientific, USA) as recommended by the manufacturer. 30 μg of protein lysates were separated in 8–10% sodium dodecyl sulfate-polyacrylamide gels for electrophoresis (SDS-PAGE)and then electrophoretically transferred to polyvinylidene difluoride (PVDF; Millipore, USA) membranes, which was then blocked with 5% non-fat milk in Tris-buffered saline with 0.1% Tween-20 (TBST)at room temperature for 1 h. Subsequently, the PVDF membranes were treated with primary antibody, including E-cadherin antibody (ab76055, Abcam, USA) at a dilution of 1:1000 and GAPDH antibody (BA2913, Boster, China) at a dilution of 1:500, overnight at 4 °C. Following extensive washing with TBST three times, membranes were further incubated with the respective secondary antibodies (Southern biotech, China) at a dilution of 1:4000 (anti-mouse) and 1:5000(anti-rabbit) at room temperature for 1 h. After further washing with TBST three times, the protein bands were detected using an Enhanced Chemiluminescence Western Blotting kit (Thermo Fisher Scientific, USA) in accordance with the manufacturer’s guidelines, and then quantified using Image Lab analysis software version 3.1.

### Dual luciferase array

Dual luciferase reporter assay was utilized to verify the target relationship between lncRNA MEG3 and miR-21, and miR-21 and E-cadherin. Firstly, for lncRNA MEG3 and miR-21, the sequences of wild-type (WT) lncRNA MEG3and mutant lncRNA MEG3were generated by PCR and then cloned into pmiR-RB-REPORORTTMVector (Ribobio, China), between the XhoI andNotI sites. Secondly, for miR-21 and E-cadherin, the sequences of WT E-cadherin and mutant E-cadherin were also amplified by PCR and inserted into the pGL3 control vector (Promega, USA). Subsequently, DNA sequencing was applied to validate these insertions. In addition, the Blank plasmid, miR-21 mimic, miR-21 inhibitor, negative control (NC) and NC inhibitor were synthetized and purchased from Sangon Biotech Co., Ltd. (Shanghai, China). B16 cells were seeded in 96-well plates and were transfected with 0.2 μg pmiR-RB-REPORORT TMVector-WT-lncRNA MEG3 or pmiR-RB-REPORORT TMVector-mutant-lncRNA MEG3, together with these purchased plasmids, or pGL3-WT-E-cadherin or pGL3-mutant-E-cadherin, together with these purchased plasmids via Lipofectamine2000 according to the manufacturer’s instructions (Invitrogen, USA). After transfecting for 48 h, the relative luciferase activity was performed by the Dual Luciferase Reporter Assay System (Promega, WI, USA).

### Statistical analysis

Results of in vitro and in vivo assays in this study were displayed as the mean ± standard deviation (SD) from three separate experiments at least. All statistical analyses were conducted using SPSS version 16.0 software and all statistical diagrams were depicted by Graph Pad Prism 6.0 software. The Student’s t-test was applied to analyze two groups for statistical significance, whereas one-way analysis of variance (ANOVA) followed by Tukey’s post hoc test was employed to analyze the difference in multiple groups (> 2). *P *< 0.05 was considered to indicate a statistically significant difference.

## Results

### lncRNA MEG3 was low expressed in melanoma tissue/cell lines and closely related to the survival rate

**Fig. 1:**
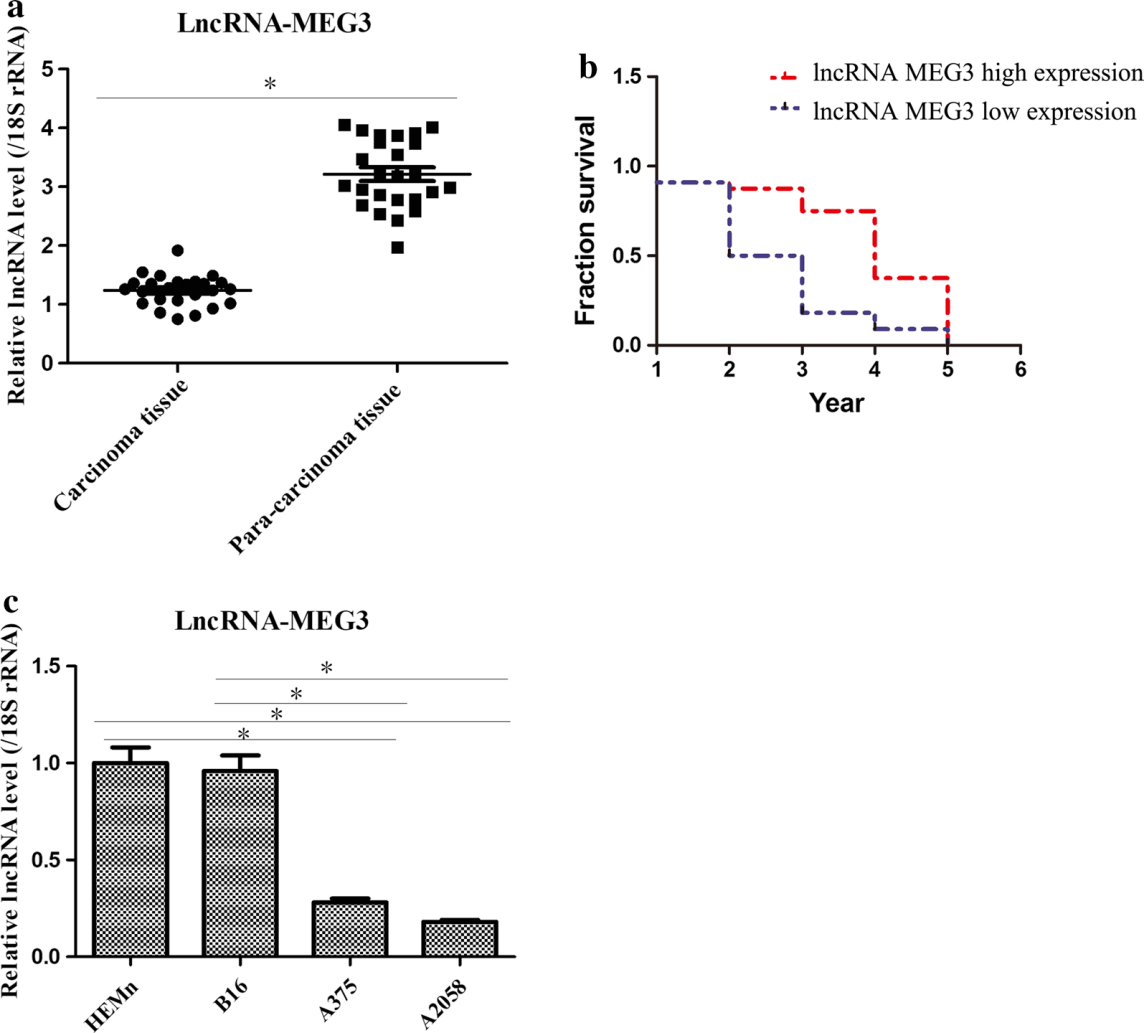
Expression of lncRNA MEG3 in melanoma tissues and cell lines. **a** RT-qPCR demonstrated that lncRNA MEG3 was low expressed in melanoma tissues after comparing 25 tumor tissues with 25 adjacent tissues. **b** The correlations between the expressions of lncRNA MEG3 and survival rate. **c** RT-qPCR showed lncRNA MEG3 expression in different melanoma cell lines

To explore the possible role of lncRNA MEG3 in melanoma, qRT-PCR was firstly conducted to compare the expression of lncRNA MEG3 in melanoma tissue with that of the adjacent tissue. The results showed that the expression of lncRNA MEG3 was lower in melanoma tissue as compared with their para-carcinoma tissues (Fig. 1a). Moreover, it was found that higher lncRNA MEG3 was closely associated with the lower survival rate (Fig. 1b). Additionally, the expression of lncRNA MEG3 was higher in B16 cells, and it also presented higher expression level in HEMn than that in A2058 and A375 cells (Fig. 1c). Therefore, these results suggested that lower expression levels of lncRNA MEG3 might be involved in the development of melanoma.

### lncRNA MEG3 suppressed the tumor growth

**Fig. 2:**
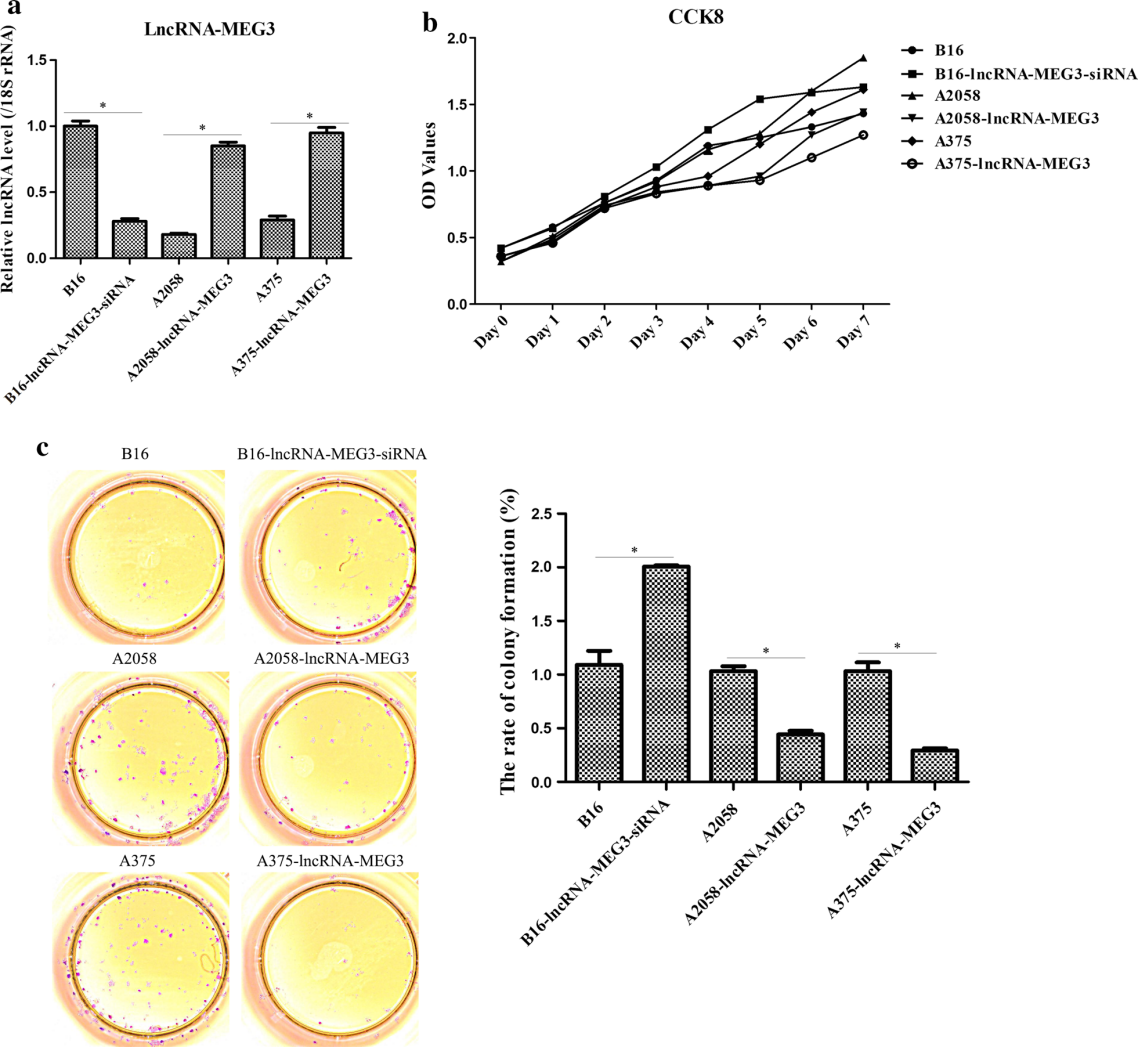
The role of lncRNA MEG3 in tumor growth of melanoma. **a** RT-qPCR was used to verify the transfected efficiency of lncRNA MEG3-siRNA and lncRNA MEG3-overexpression plasmids. **b** CCK8 assay was utilized to examine the cell proliferation of melanoma cells. **c** Colony formation assay was applied to evaluate the cell proliferation of melanoma cells

Based on the different expression of lncRNA MEG3 in human melanoma cell lines, we chose B16 (higher lncRNA MEG3 expression), A2058 (lower lncRNA MEG3 expression) and A375 (lower lncRNA MEG3 expression) cells for the following experiments. Before describing the follow-up results, we hereby make a statement. We chose B16 (mouse cell line) and A2058/A375 (human cell line) not to compare the difference between mouse cell line and human cell line, but only because lncRNA MEG3 was highly expressed in mouse cell lines and low in human cell lines, and we wanted to investigate the biological role of high and low expression of lncRNA in melanoma cells, regardless of cell lineage differences. At first, lncRNA MEG3-siRNA was transfected into B16 cells, while lncRNA MEG3-overexpression plasmid was transfected into A2058 and A375 cells. The transfected efficiency was determined by qRT-PCR which exhibited that lncRNA MEG3 expression was decreased in lncRNA MEG3-siRNA treated B16 cells, and lncRNA MEG3 expression was increased in lncRNA MEG3 treated A2058 or A375 cells (Fig. 2a). Then, CCK8 assay was used to observe the cell proliferation. It was found that lower lncRNA MEG3 expression in B16 cells gradually promoted cell proliferation, but higher lncRNA MEG3 expression in A2058 or A375 cells gradually impeded cell proliferation (Fig. 2b). Furthermore, the colony formation assay, which also represented cell growth indexes, presented a similar trend with CCK8 assay in different groups (Fig. 2c). Thus, these data indicated that lncRNA MEG3 transfected B16 cells had more obvious stimulative effect on melanoma cell proliferation than lncRNA MEG3-siRNA transfected A2058 and A375 cells.

### lncRNA MEG3 inhibited the tumor metastasis and formation

**Fig. 3:**
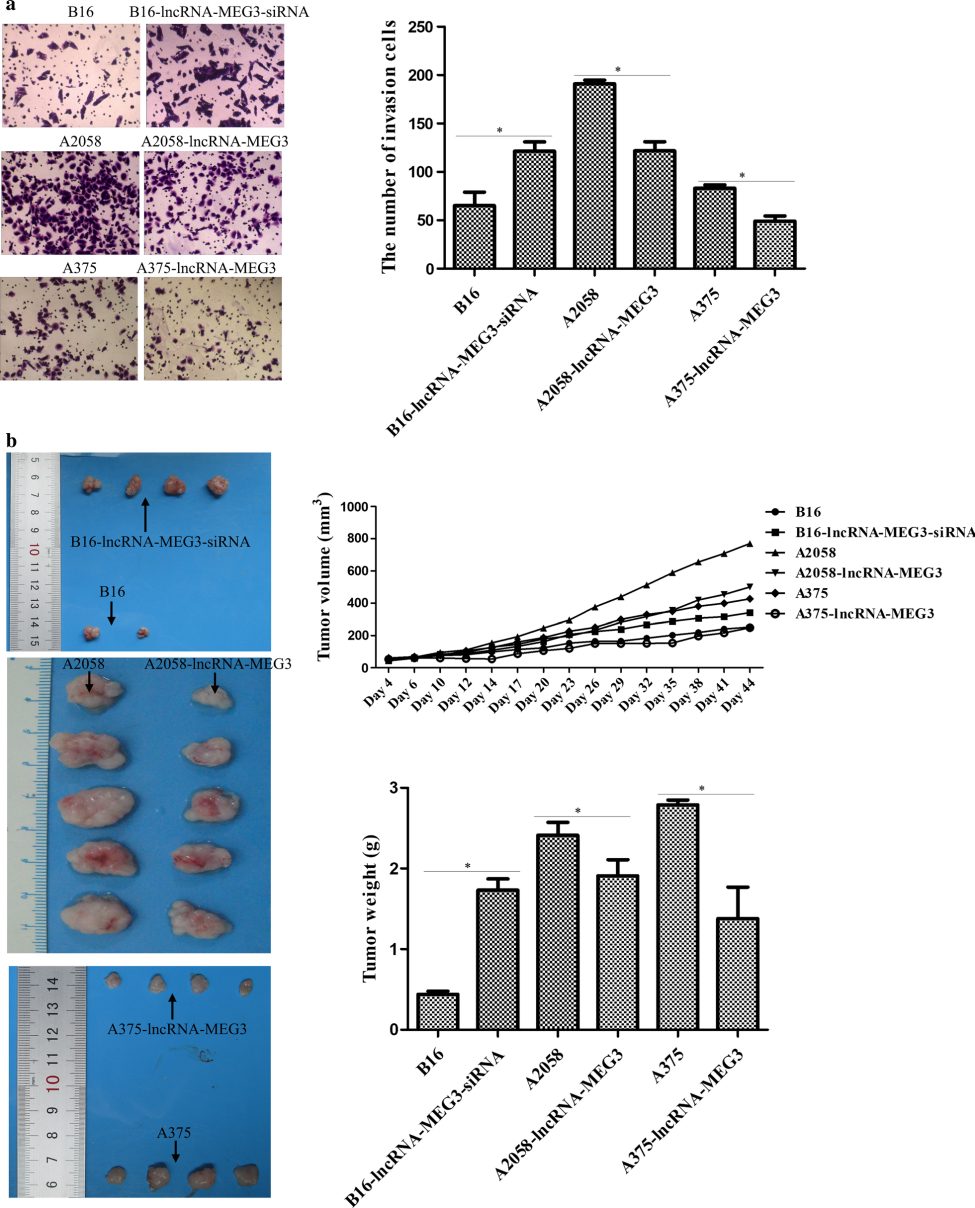
The role of lncRNA MEG3 in tumor metastasis and formation of melanoma. **a** Cell migratory ability was determined by transwell assay. **b** The progression of tumor generation was assessed by tumor xenografts in nude mice

In order to further investigate the biological functions of lncRNA MEG3, cell migration assay and tumor xenografts in nude mice were conducted. It was discovered that cell migration was elevated dramatically by lncRNA MEG3 knockdown in B16 cells, while cell migration was conspicuously reduced by lncRNA MEG3 overexpression in A2058 and A375 cells when compared with respective controls (Fig. 3a). In addition, in the experiment of tumor xenografts, it was displayed that in comparisons with B16 group, the tumor volume was substantially elevated in B16-lncRNA MEG3-siRNA group, but the tumor volume in A2058 or A375 group was notably lighter than that in A2058-lncRNA MEG3 or A375-lncRNA MEG3 group, respectively (Fig. 3b). As well, after 44 days, all nude mice were sacrificed for tumor weight examination and the results revealed that the tumor weight in B16-lncRNA MEG3-siRNAgroup was greater than that in B16group, whereas the tumor weight was sharply decreased in A2058-lncRNA MEG3 or A375-lncRNA MEG3 group as compared to their corresponding control groups (Fig. 3b). Hence, these findings implied that lncRNA MEG3 suppression might enhance the tumor metastasis and formation of melanoma cell.

### E-cadherin was significantly down-regulated in melanoma patients and cell lines

**Fig. 4:**
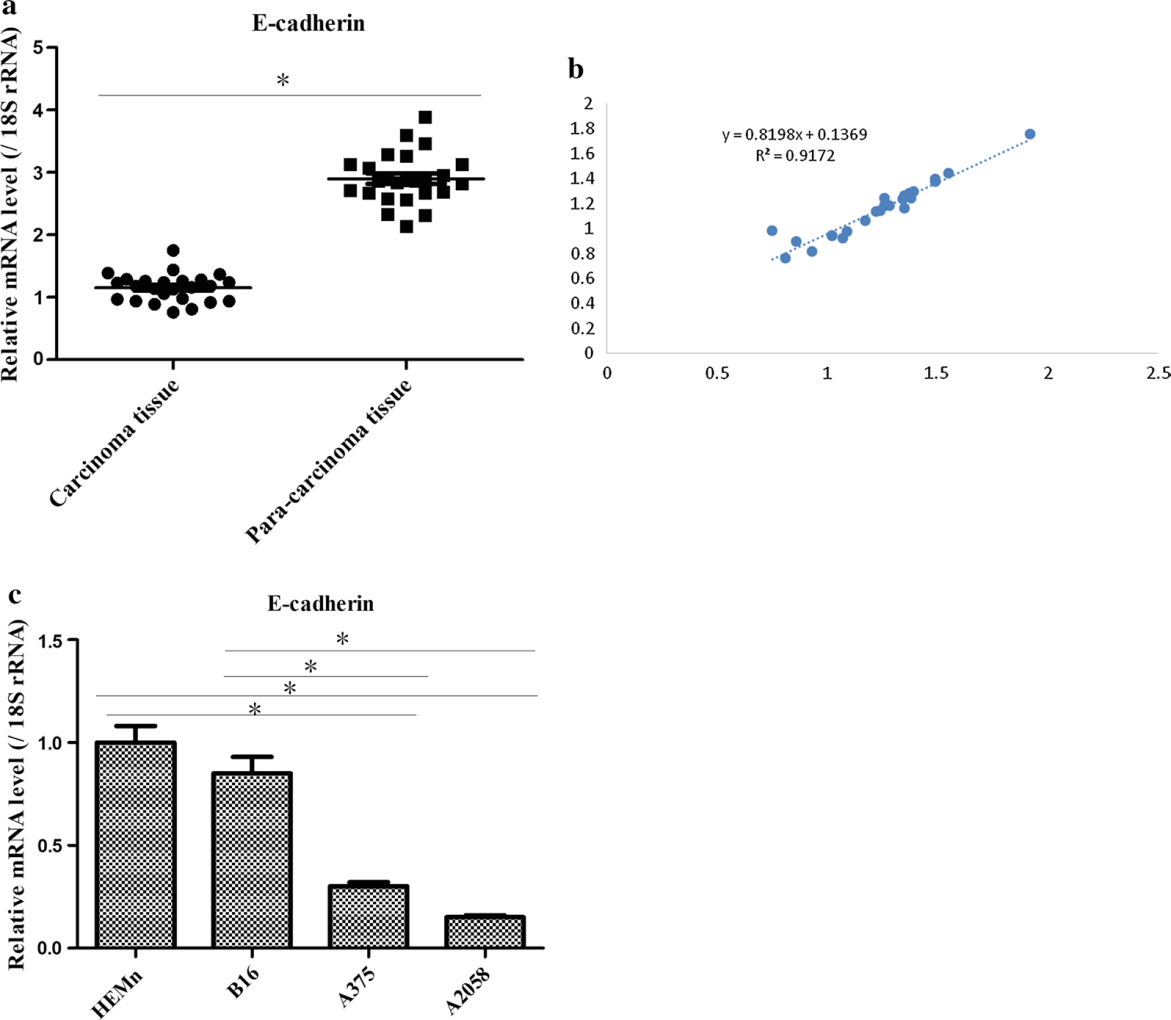
Expression of E-cadherin in melanoma tissues and cell lines. **a** RT-qPCR revealed that E-cadherin mRNA levels in melanoma tissues were lower than that in the corresponding adjacent tissues. **b** Kaplan–Meier curves were constructed to demonstrate the correlation between lncRNA MEG3 and E-cadherin. **c** RT-qPCR displayed E-cadherin expression in different melanoma cell lines

qRT-PCR data demonstrated that E-cadherin was clearly declined in melanoma tumor tissues compared with the adjacent non-tumor tissues (Fig. 4a). Moreover, there was a positive correlation property between E-cadherin and lncRNA MEG3 (Fig. 4b). Additionally, as similar with the expression of lncRNA MEG3 in melanoma cell lines, it was uncovered that the expression of E-cadherin was higher in HEMn and B16 cells than that in A2058 and A375 cells (Fig. 4c). Thereby, these results hinted that the E-cadherin expression might play an important role which was intimately related to lncRNA MEG3 in progression of melanoma.

### Knockdown of E-cadherin accelerated tumor growth, metastasis and formation

**Fig. 5:**
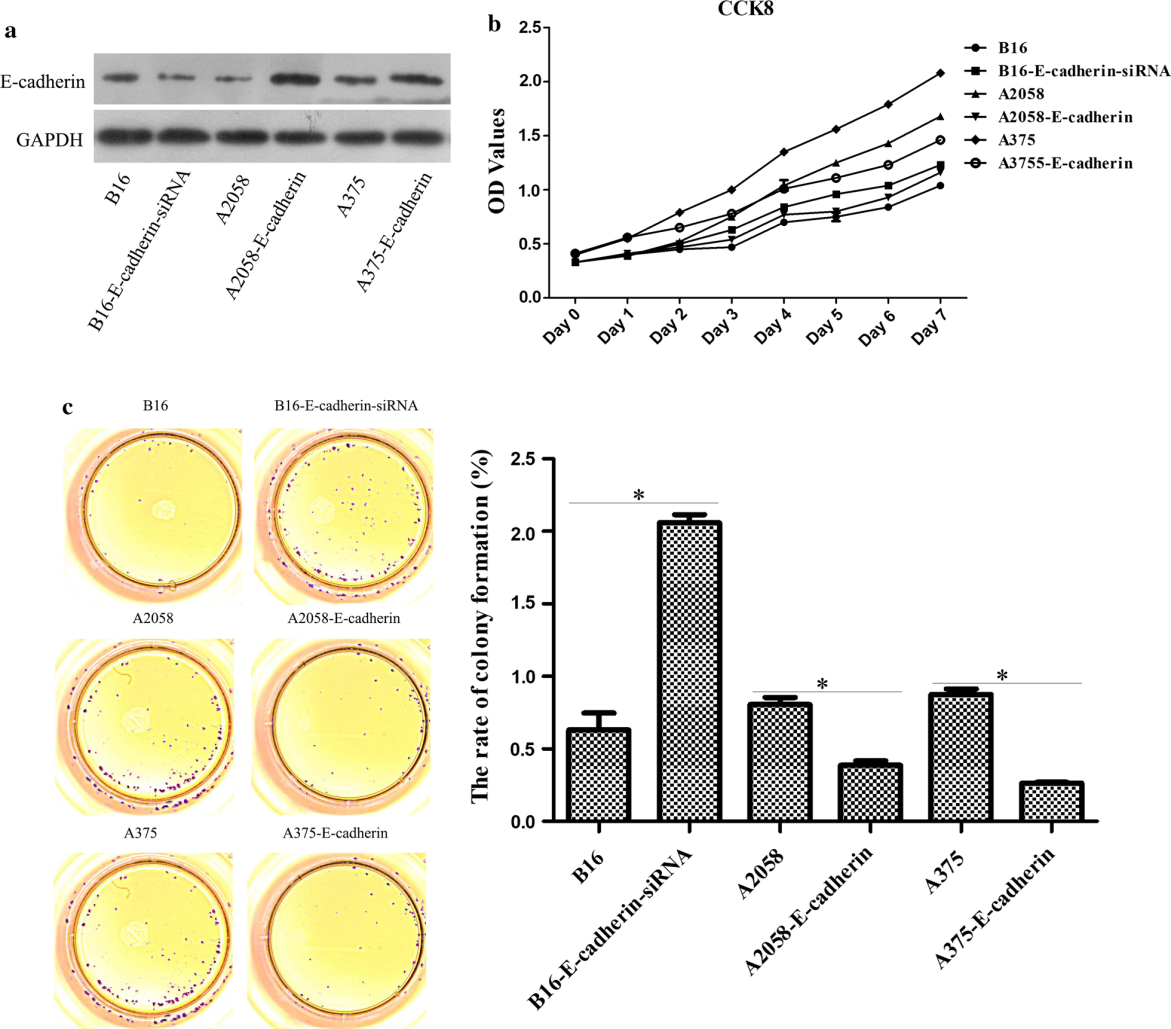
The effects of E-cadherin in tumor growth of melanoma. **a** RT-qPCR was used to verify the transfected efficiency of E-cadherin-siRNA and E-cadherin-overexpression plasmids. **b** CCK8 assay was utilized to examine the cell proliferation of melanoma cells. **c** Colony formation assay was applied to evaluate the cell proliferation of melanoma cells

**Fig. 6:**
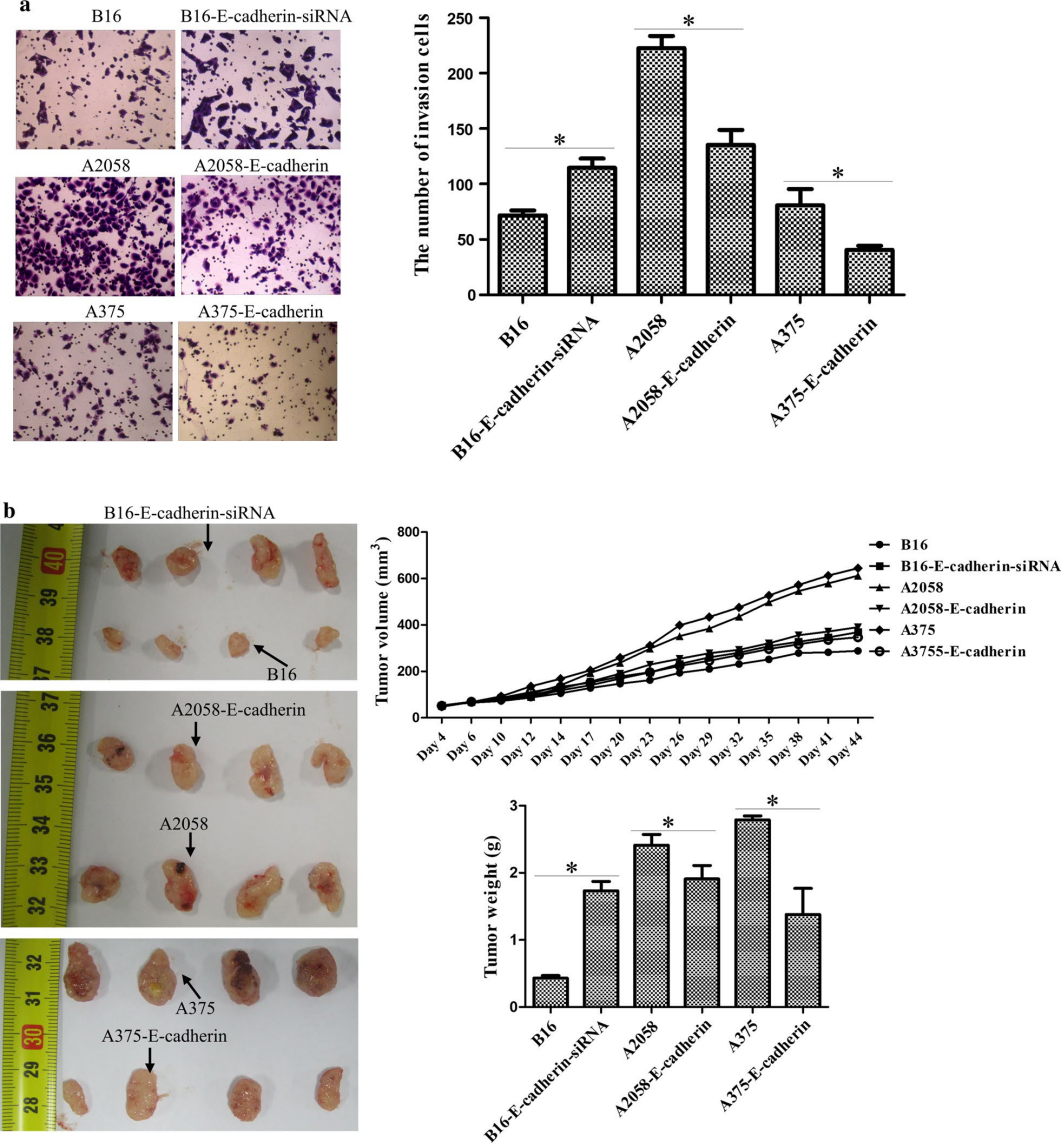
The role of E-cadherin in tumor metastasis and formation of melanoma. **a** Cell migratory ability was determined by transwell assay. **b** The progression of tumor generation was assessed by tumor xenografts in nude mice

According to the data from qRT-PCR which measured the expression of E-cadherin, we selected B16 (higher E-cadherin expression), A2058 (lower E-cadherin expression) and A375 (lower E-cadherin expression) cells for the subsequent study. Stable E-cadherin-siRNA transfected B16 cells and stable E-cadherin transfected A2058 and A375 cells were confirmed by WB (Fig. 5a). Next, these stable cell lines were applied to determine the biological functions of E-cadherin. CCK8 assay and colony formation assay both verified that knockdown of E-cadherin markedly elevated the B16 cell growth, and overexpression of E-cadherin remarkably reduced the A2058 and A375 cell growth (Fig. 5b, c). In addition, transwell analysis pointed out that the migratory capacity of E-cadherin-siRNA B16 cells was enhanced compared with that of the B16 group, whereas the overexpression of E-cadherin expression decreased the migratory capacity of the A2058 and A375 cells as compared to their respective controls (Fig. 6a). Furthermore, we established an in vivo pretreated xenograft nude mouse model to further explore the role of E-cadherin. As illustrated in Fig. 6b, compared to B16 group, depletion of E-cadherin led to a quick tumor growth in vivo, but when compared with A2058 and A375 groups, forced E-cadherin expression resulted in obvious retardation of tumor growth in vivo. After 44 d, E-cadherin-siRNA transfected B16 cells produced larger tumors than those in B16 cells, whereas E-cadherin-transfected A2058 and A375 cells produced smaller tumors than those in A2058 and A375 groups. The above results were in accordance with these findings in the melanoma cell lines treated with lncRNA MEG3-overexpression and lncRNA MEG3-siRNA plasmids. Collectively, these data concluded that there might be a suppressive effect of higher E-cadherin expression in tumor growth, metastasis and formation of melanoma.

### miR-21 was a target gene of lncRNA MEG3and E-cadherin was a target gene of miR-21

**Fig. 7:**
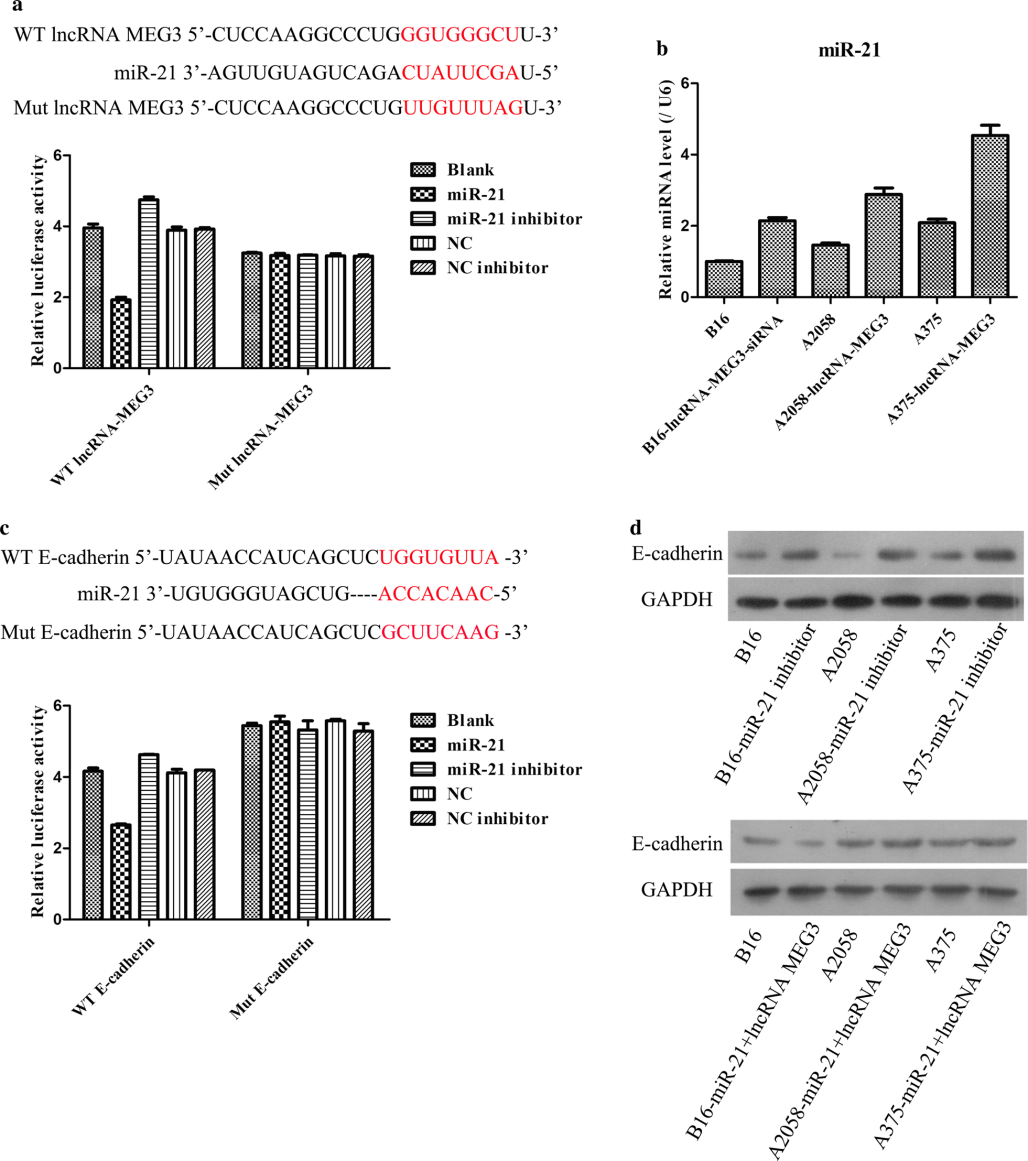
The confirmation of relationship among lncRNA MEG3, miR-21 and E-cadherin. **a** Dual luciferase reporter assay was conducted to test the interaction between lncRNA MEG3 and miR-21. **b** miR-21 expression was monitored in melanoma cells treated with lncRNA MEG3-siRNA and lncRNA MEG3-overexpression plasmids using RT-qPCR. **c** Dual luciferase reporter assay was performed to detect the interaction between miR-21 andE-cadherin. **d** E-cadherin expression was checked in melanoma cells treated with miR-21 + lncRNA MEG3 via WB

As presented in Fig. 7a, c, dual luciferase assays directly revealed that miR-21 led to a marked reduce in luciferase activity in lncRNA MEG3-WT reporter compared with Blank group, but had no obvious effect on the luciferase activity in lncRNA MEG3-mutant reporter; and meanwhile miR-21also resulted in a distinct reduce in luciferase activity in E-cadherin-WT reporter compared with Blank group, but had no obvious effect on the luciferase activity in E-cadherin-mutant reporter, which manifested that lncRNA MEG3 could target miR-21 and miR-21 could target E-cadherin. Moreover, when changing the expression of lncRNA MEG3 and miR-21 in melanoma cell lines, it was found that miR-21 and E-cadherin both presented a negative alteration, respectively (Fig. 7b, d). Taken together, these findings disclosed that there might be a regulatory axis related to lncRNA MEG3/miR-21/E-cadherin.

## Discussion

Melanoma is a cutaneous cancer caused by the malignant transformation of pigment-producing cells melanocytes located predominantly in the skin and characterized by high metastatic potentials [23]. Moreover, it is responsible for 75–80% of deaths from skin cancer and generally higher in fair-skinned population [24]. In recent years, there were still no satisfactory treatments to fight melanoma, especially for the advanced patients [25]. Accumulating evidence revealed that the etiology of melanoma involved environmental, phenotypic and genetic risk factors, so a large number of researchers began to pay their attentions to the molecular level, in order to find useful biomarkers for non-invasive early detection or new effective therapeutic targets in melanoma patients [23]. lncRNAs, historically dismissed as junk or nonfunctional transcriptional noise, play crucial roles in different cancers through modulating gene expression with various mechanisms [26]. lncRNA MEG3, located in the chromosome 14 DLK1-MEG3 imprinting region, were down-regulated in a variety of primary human cancers, including lung cancer, hepatocellular cancer, multiple myeloma, meningioma and glioma [27]. In the current study, it was also discovered that lncRNA MEG3 presented a declining trend in carcinoma tissues as compared with their para-carcinoma tissues of melanoma. Moreover, the expression level of lncRNA MEG3 was intimately associated with the survival rate of melanoma, which suggested that lncRNA MEG3 might be served as a prognostic indicator in development of melanoma. Factually, it has been reported that lncRNA MEG3 expression level correlated with tumor grade and prognosis in meningiomas [28]. In order to further excavate the functions of lncRNA MEG3, we examined the expression of lncRNA MEG3 in different melanoma cell lines and finally selected 3 strains of melanoma cell lines for the following study. The results showed that downregulation of the expression of the lncRNA MEG3 promoted melanoma growth, metastasis and formation; thereby it speculated that lncRNA MEG3 might exert a tumor suppressor role in development of melanoma. Additionally, mounting studies also verified that lncRNA MEG3 inhibited tumor initiation and progression [15]. For example, lncRNA MEG3 inhibits breast cancer growth via upregulating endoplasmic reticulum stress and activating NF-κB and p53 [29]; lncRNA MEG3 impacts proliferation, invasion, and migration of ovarian cancer cells through regulating PTEN [30].

E-cadherin has been implicated in a number of signaling pathways that enhance cell–cell adhesion and cell–cell interactions [31]. Once the cell-to-cell junction is destroyed, it may lead to the metastasis of tumor cells [32]. Nevertheless, metastasis of tumor cells is a significant marker of tumor progression, which indicates that the tumor has reached an irreversible level and is difficult to treat [33]. Thus, in this study, we subsequently detected the expression of E-cadherin in melanoma patients and cell lines. It was exhibited that E-cadherin expression in carcinoma tissues was notably lower than that in their para-carcinoma tissues. Furthermore, based on the different expressions of E-cadherin in melanoma cell lines, it was further investigated the biological effects of E-cadherin in vitro. The data displayed that knockdown of E-cadherin accelerated melanoma tumor growth, metastasis and formation. The above results related to E-cadherin were amazingly similar with the results of lncRNA MEG3 treatment. Hence, we boldly guessed whether there is a potential relationship between lncRNA MEG3 and E-cadherin. Moreover, the analysis of Kaplan–Meier curves indeed manifested that there was a positive correlation between lncRNA MEG3 and E-cadherin. Fortunately, we have also found a key link molecule (that is miR-21) between lncRNA MEG3 and E-cadherin through bioinformatics screening.

Previous study has confirmed that miR-21 was over-expressed in primary cutaneous melanomas when compared with benign nevi, and was also highly expressed in melanoma cells [34]. In addition, high levels of miR-21 are correlated with advanced tumor stage, degree of invasion and tumor recurrence of melanoma patients, which mainly due to miR-21 could inhibit mRNA expression of crucial tumor suppressor proteins; thereby miR-21 acted as a key oncogene in malignant melanoma [20]. In the present study, dual luciferase reporter assay firstly used to determine the interaction between lncRNA MEG3 and miR-21. And the data clearly denoted that miR-21 was a target gene of lncRNA MEG3. Over the past decades, lncRNAs functioned as competing endogenous (ce)RNAs to sponge miRNAs and regulated their downstream signaling pathways is a research hotspot [26]. On the other hand, the target relationship between miR-21 and E-cadherin was also detected by dual luciferase reporter assay. Thus, it was speculated that lncRNA MEG3 restrained the expression of miR-21 via sponging miR-21, and meanwhile down-regulation of miR-21 could promote the expression level of downstream target gene E-cadherin. This assumption meets well with the above results about the expressions of lncRNA MEG3 and E-cadherin in clinical samples. In addition, the finally WB results of E-cadherin definitely disclosed that when giving miR-21 inhibitor in lncRNA MEG3 higher expressed B16 cells, the E-cadherin expression was remarkably increased, but when giving miR-21 + lncRNA MEG3 in lncRNA MEG3 higher expressed B16 cells, the E-cadherin expression was markedly decreased. Furthermore, in lncRNA MEG3 lower expressed A2058 and A375 cells, the expression of E-cadherin presented an opposite trend. These data further proved the targeted regulatory interaction among lncRNA MEG3, miR-21 and E-cadherin.

## Conclusion

In conclusion, this report emphasizes that lncRNA MEG3 acts as an antitumor lncRNA for malignant melanoma by regulating miR-21/E-cadherin axis. Lower expressions of lncRNA MEG3 and E-cadherin were both able to enhance the melanoma tumor growth, metastasis and formation. miR-21 is an important link between lncRNA MEG3 and E-cadherin. Collectively, the present study helped to reveal that targeting lncRNA MEG3/miR-21/E-cadherin axis may be a promising therapy strategy for melanoma patients. Meanwhile, because of the defects in in vitro experiments, more mechanistic researches and animal experiments are required for better elaborating the detailed role of lncRNA MEG3/miR-21/E-cadherin axis in melanoma.

## Data Availability

The datasets during and/or analyzed during the current study available from the corresponding author on reasonable request.

## Abbreviations

LnRNA: long non-coding RNA
MEG3: maternally expressed gene 3
SPHK1: sphingosine kinase 1
CCK: Cell Counting Kit
RIPA: radioimmunoprecipitation assay
SDS-PAGE: sodium dodecyl sulfate-polyacrylamide gels for electrophoresis
PVDF: polyvinylidene difluoride
WT: wild-type
NC: negative control
SD: standard deviation
ANOVA: analysis of variance
