# Correction to: LncRNA MEG3 promotes melanoma growth, metastasis and formation through modulating miR-21/E-cadherin axis

**DOI:** 10.1186/s12935-020-01239-2

**Published:** 2020-05-11

**Authors:** Liangcai Wu, Lifei Zhu, Yanchang Li, Zhixin Zheng, Xi Lin, Chaoying Yang

**Affiliations:** 1 Department of Dermatology, The Sixth Affiliated Hospital, Sun Yat-sen University, No. 26, Yuancun ErHeng Road, Guangzhou, 510655 China; 2 Department of Pharmacology, Medical College, Jinan University, Guangzhou, 510632 China

## Correction to: Cancer Cell Int (2020) 20:12 10.1186/s12935-019-1087-4

Following publication of the original article [1], we were notified of a mistake in the article’s title. The title should read “inhibits” instead of “promotes”, as follows: “LncRNA MEG3 inhibits melanoma growth, metastasis and formation through modulating miR-21/E-cadherin axis”.
